# Infection and Disinfection

**Published:** 1887-04-30

**Authors:** 


### Till the Doctor Comes.

[Inquiries and Communications for this column should be addressed, "Physician," care of the Editor of THE HOSPITAL.]

### XVII.—INFECTION AND DISINFECTION.

THERE are certain diseases which are known to be infectious, that is, able to be communicated from one person to another, either by direct contact, through the medium of the atmosphere, or otherwise. The present remarks will apply to the acute infectious diseases commonly termed zymotic diseases. The following is a list of them :—

| | |
|---|---|
| Chicken-pox. | Measles. |
| Cholera. | Puerperal Fever. |
| Diphtheria. | Pyæmia. |
| Enteric, or Typhoid Fever. | Relapsing Fever. |
| | Scarlatina, or Scarlet Fever. |
| Erysipelas. | Small-pox. |
| Typhus Fever. | Whooping-cough. |

A person suffering from any one of these diseases should be thoroughly isolated from all other members of the family. A room should be prepared for him, by removing all superfluous hangings, carpets, curtains, pictures, etc. ; and two persons should be told off to wait on him, who should not go near any others. Great attention should be paid to the ventilation of the room ; and if care be taken to prevent draughts from blowing directly on the patient, not the least fear need be felt in giving very free ventilation. There should be a little fire in the room to promote free movement of air. In the same way, too, there need be no hesitation in washing a patient daily, with the precautions we have before indicated; in fact, copious ablution is, in infectious cases, most essential, though it has unfortunately come to be considered that ventilation and washing in such cases is prejudicial. This notion cannot be too emphatically condemned. A little Condy's fluid in the water is an advantage. Perfect cleanliness must be enforced in the sick-room ; and all slops, foul linen, etc., must have some disinfectant poured on them, and be immediately removed from the room. A sheet hanging outside the door of the room, and kept wet with a disinfectant solution, is useful to prevent the spread of the disorder. The bed should be placed in the centre of the room, and, if necessary, a screen arranged so as to keep off any draughts or glare of light. It cannot be enforced too strongly, that there are no means of cutting short these fevers ; they run a stated course, and the object in treatment is to nurse the patient carefully through such course, keeping a vigilant watch, and trying to prevent the different complications peculiar to each of them. During the course of the disease, solid food, if taken, is not usually digested, and therefore must not be given to the patient except under medical instructions. Milk and beef-tea will be his almost sole support, combined with stimulants, under the guidance of the doctor. Cold water is generally most earnestly begged, and it may certainly be allowed, if too much is not taken at any one time. The person acting as nurse must be careful not to bend over the patient and inhale his breath, and also not to meddle with any of the foul linen and other matters more than is absolutely necessary. A walk in the fresh air once a day is also very desirable for her. Should the patient become delirious, do not employ strait-waistcoats, do not talk to the patient, nor contradict him, and, above all, do not wrestle with him. All these things are quite unnecessary. If the nurse is calm and gentle, and knows how to manage her patient, a little tact is all that is required. She should, however, never lose sight of him for an instant; and all dangerous weapons should be removed. No sponges must be used, and, as far as practicable, everything that can be burnt after use must be so treated, such as poultices, dressings, etc. No woollen garments should be worn in the room, but dresses of cotton, or some material that will wash. There are certain public duties which ought to be performed in connection with this class of disease. Thus in some towns it is compulsory, and in all cases it ought to be the rule, that the medical officer of health or inspector of nuisances should be immediately made acquainted with any case of infectious disease. One of these officers would then, if necessary, visit the house, and give directions, disinfectants, etc., that would tend to limit the spread of the disease ; but it need not be feared that he will interfere with the proper duties of the medical attendant. Again, if a patient has to be removed from one place to another, and there is no proper ambulance for the purpose, notice must be given to the cabman as to the nature of the case, and he must be paid such reasonable sum, in addition to his fare, as will enable him to disinfect his cab. If he do not do so he is liable to be fined heavily.

In *Scarlet Fever* and *Small-pox*, the scales and dusty powder escaping from the skin are highly infectious. To prevent their diffusion throughout the room, it is advisable to smear the body of the patient daily with camphorated oil, or, better still, with carbolic oil (made by adding one part of pure carbolic acid to forty or sixty parts of olive oil). In measles there is a great tendency for bronchitis and other lung complaints to develop. More care must therefore be taken to protect the patient from draughts, and the temperature of the room may be kept a little higher.

*Typhus Fever.*—This is very different from typhoid. It is a great pity that the names are so similar, and, consequently, so often mistaken. More often typhoid is now called enteric fever, and it will be well if this name is universally adopted. Typhus fever is exceedingly infectious, and demands free ventilation in its treatment.

